# On reporting results from randomized controlled trials with recurrent events

**DOI:** 10.1186/1471-2288-8-35

**Published:** 2008-05-30

**Authors:** Lisa Kuramoto, Boris G Sobolev, Meghan G Donaldson

**Affiliations:** 1 Centre for Clinical Epidemiology and Evaluation, Vancouver Coastal Health Research Institute, Vancouver, Canada; 2 Department of Health Care and Epidemiology, University of British Columbia, Vancouver, Canada; 3 SF Coordinating Center, San Francisco, USA

## Abstract

**Background:**

Evidence-based medicine has been advanced by the use of standards for reporting the design and methodology of randomized controlled trials (RCT). Indeed, without this information it is difficult to assess the quality of evidence from an RCT. Although a variety of statistical methods are available for the analysis of recurrent events, reporting the effect of an intervention on outcomes that recur is an area that remains poorly understood in clinical research. The purpose of this paper is to outline guidelines for reporting results from RCTs where the outcome of interest is a recurrent event.

**Methods:**

We used a simulation study to relate an event process and results from analyses of the gamma-Poisson, independent-increment, conditional, and marginal Cox models. We reviewed the utility of regression models for the rate of a recurrent event by articulating the associated study questions, preenting the risk sets, and interpreting the regression coefficients.

**Results:**

Based on a single data set produced by simulation, we reported and contrasted results from statistical methods for evaluating treatment effect from an RCT with a recurrent outcome. We showed that each model has different study questions, assumptions, risk sets, and rate ratio interpretation, and so inferences should consider the appropriateness of the model for the RCT.

**Conclusion:**

Our guidelines for reporting results from an RCT involving a recurrent event suggest that the study question and the objectives of the trial, such as assessing comparable groups and estimating effect size, should determine the statistical methods. The guidelines should allow clinical researchers to report appropriate measures from an RCT for understanding the effect of intervention on the occurrence of a recurrent event.

## Background

Evidence-based medicine has been advanced by the use of standards for reporting the design and methodology of randomized controlled trials (RCT). Indeed, without this information it is difficult to assess the quality of evidence from an RCT. An increasing number of journals demand that submissions adhere to the Consolidated Standards for Reporting Trials (CONSORT) guidelines for improving report quality [1]. However, there are not yet available guidelines for reporting results from RCTs in which the subject may experience the same event multiple times during follow-up. Examples of recurrent events include falls, fractures, certain cancers, infections, chronic disease exacerbations, and hospitalizations [2-7]. Through a trial, clinical researchers attempt to determine whether the study outcome occurs more frequently in the intervention group than in the control group. In such trials clinicians are interested in a variety of questions, such as "How many events does the intervention prevent, on average, compared to the control?"; "Does the intervention decrease the event rate over the study period compared to the control?"; "What is the effect of intervention on the rate of subsequent event among those who experienced the preceding event?"; and "What is the protective effect of intervention on the rate of higher-order events compared to the control?"

Although a variety of statistical methods are available for the analysis of recurrent events, reporting the effect of an intervention on outcomes that recur is an area that remains poorly understood in clinical research [8,9]. Appropriate statistical techniques are not always used to analyze RCTs on recurrent falls [9]. Extensive work involving simulation studies based on varying event processes and case studies have compared recurrent event methods to illustrate their strengths and weaknesses [10-13]. Such methods include the gamma-Poisson model, and several extensions of the Cox proportional hazards model, including the independent-increment, marginal, and conditional models [14-20].

The purpose of this paper is to outline guidelines for reporting results from a trial of treatment that prevents a recurrent event. As an example, we are using the rationale of a randomized trial on falls prevention. Falls are the most common cause of injury among elderly people. One in three persons over the age of 65 falls at least once each year and this proportion increases to one in two people over the age of 80 [21,22]. Almost half of those who fall experience the event recurrently [23,24]. The goal of RCTs is to reduce the occurrence of falls with specific interventions strategies such as multi-factorial intervention, strength and balance retraining, medication rationalization and expedited cataract surgery.

In the Methods section we review the utility of regression models for the rate of a recurrent event by articulating the associated study questions, presenting the risk sets, and interpreting the regression coefficients. Based on a single data set produced by simulation, we report and contrast results from statistical methods for evaluating treatment effect from an RCT with a recurrent outcome in the Results section. Finally, we summarize our guidelines for reporting evidence from RCTs on recurrent events.

## Methods

In this section, we relate study questions of interest in RCTs to methods for modelling recurrent event data. Recurrent event models were developed to account for potential dependence among observations within a subject. One approach allows for unobserved heterogeneity which is unmeasured, intraclass correlation where subjects have constant but unequal probabilities of experiencing the event [25]. Three other models, which were developed for the analysis of continuous time recurrent event data, are extensions of the Cox proportional hazards model. They first fit a Cox model that ignores dependence and then use the empirical sandwich estimator to adjust standard errors for the parameter estimates [17,18,20]. Several authors argued for a conditional approach that estimates the rate of *k*th event among those who have already experienced (*k *- 1) events [18,26]. This approach addresses the issue of constant susceptibility in a more natural way than marginal models [18,27]: while the association between event times remains unspecified, the event-specific rate functions condition on having had previous events.

There are substantial differences among the models described in this section, but all estimate the effect of factors on the occurrence and time to event while accounting for the dependence between observations. The methods that we review model the rate function, *λ*(*t*)-that is, the average intensity of a recurrent event at a certain time. We highlight differences in the model assumptions, risk sets, and rate ratio interpretation. The data structure required to fit each model is shown to illustrate the different risk sets, indicating which patients are considered to be at risk for events at certain times [25,28]. Examples of SAS code (SAS System version 9.1 for Windows, SAS Institute Incorporation, Cary, NC, USA) to fit each model are also presented.

### Mean cumulative function

"How many events does the intervention prevent, on average, compared to the control?" is one study question in an RCT on recurrent events that could be addressed using the mean cumulative function (MCF). The MCF shows the population mean number of recurrent events by certain times [29]:

MCF(*t*) = E{*N*(*t*)}.

where *N*(*t*) is a random variable for the number of events that have occurred up to time *t*. The MCF curve changes as a function of time and its derivative gives the rate function, that is

$$\lambda (t)=\frac{d}{dt}\text{MCF}(t).$$

The rate and intensity functions quantify different aspects of the recurrent event process: the intensity is the instantaneous risk of a recurrent event and the rate is the average intensity at time *t *[25,30]

*λ*(*t*)*dt *= E[*dN*(*t*)],

where *dN*(*t*) denotes the number of events in a small interval [*t, t *+ *dt*).

We interpret the difference in MCFs between the intervention and control groups as an indicator of how many events the intervention would prevent, on average, by a certain time [31].

### Gamma-Poisson model

A common study question for an RCT on recurrent events is "Does the intervention decrease the event rate over the study period compared to the control?", for which the gamma-Poisson model has been used. The gamma-Poisson model evaluates the relationship between the number of recurrent events and factors of interest when the data deviate from the Poisson model [15,16]. This model allows variation of the event rate among subjects in the same group according to an unobserved random variable, frailty, which defines how likely a subject is to experience the event compared to the average rate [16]. When the frailty follows a gamma distribution and a time homogeneous model is assumed then the marginal distribution of the total number of events is negative binomial [15].

Suppose *N*_*i*_(*t*) counts the number of events that have occurred up to time *t *for subject *i*. Under the time-homogeneous, gamma-Poisson model, *N*_*i*_(*t*) has a Poisson distribution with rate function

(1) *λ*_*i*_(*t*) = *μ*_*i *_exp{*α*_0 _+ *βx*_*i*_},

where *μ*_*i *_come from a gamma distribution with density function

(2) $$f(\mu )=\frac{\mu^{1/\theta -1}\exp(-\mu /\theta )}{\Gamma (1/\theta )\theta^{1/\theta }}.$$

In model 1, *α*_0 _is the logarithm of the baseline rate for the event, *μ*_*i *_is the unobserved frailty for subject *i*, *x*_*i *_is a covariate value for subject *i*, *β *is the regression coefficient, and *t *represents the time from start of observation.

The expected value and variance of the frailty random variable is 1 and *θ*, respectively. Subjects with *μ*_*i *_greater than 1 are considered more "frail" or more likely to experience the event at a higher rate; whereas, those with *μ*_*i *_less than 1 are considered to experience the event at a lower rate [16].

Compared to the Poisson model which assumes the mean and variance for the number of events are equal, the gamma-Poisson model has an additional parameter which allows for over-dispersion. For a given set of covariates, this model assumes the expected number of events is *t *exp(*α*_0 _+ *βx*_*i*_) and the variance is *t *exp(*α*_0 _+ *βx*_*i*_) + *θt*^2 ^exp(*α*_0 _+ *βx*_*i*_)^2 ^[32].

The rate function of any event for subject *i *averaged over the gamma-distribution is

(3) $${\bar{\lambda }}_{i}(t)=\exp\{\alpha_{0}+\beta x_{i}\}{\left[1+\theta t\exp\{\alpha_{0}+\beta x_{i}\}\right]}^{-1}.$$

Subjects are at risk of an event until they are censored. Suppose *x*_*i *_is a binary indicator of group membership, with value 0 if subject *i *belongs to the control group and 1 if the intervention group. Then, exp $\left(\hat{\beta }\right)$ from model 3 estimates the common rate ratio of event in the intervention group relative to the control. We interpret rate ratios less than 1 as indicating the overall rate of event, that is the rate of any event, in the intervention group is 100 [1 - exp$\left(\hat{\beta }\right)$]% lower than in the control.

The data structure for this model requires one record for each subject, regardless of the number events experienced. This record contains the total follow-up time and total number of events per subject. The data structure required for this model is illustrated through an example. Suppose subject 1 in the control group experiences a recurrent event at day 126, 216, and 314 from study start and is followed up for 365 days. In addition, subject 2 in the intervention group, who was followed for the same period of time, had events at day 42 and 350. Under the time-homogeneous gamma-Poisson model, the data for these subjects are represented as shown in Table 1. In this data set, pid is the subject identifier, time is the total follow-up time, nevent is the total number of events experienced, grp is the covariate for group membership, and logtime is the natural logarithm of time.

**Table 1:** Data structure for the time-homogeneous gamma-Poisson model

pid	time	nevent	grp	logtime
1	365	3	0	5.899
2	365	2	1	5.899

For these data, SAS can be used to fit a time-homogeneous gamma-Poisson model:

PROC GENMOD;

   MODEL nevent = grp/LINK = LOG DIST = NEGBIN OFFSET = logtime;

RUN;

A major limitation of the time-homogeneous gamma-Poisson model is it assumes that the recurrent event rate is constant over time, which is unlikely to hold in practice. Extensions to this model have been made to relax the independent increment assumption for recurrent events and the specification of the within subject correlation between recurrence times. For example, the general frailty model assumes that the counting process is a non-homogeneous Poisson process given the frailty and covariates, where the frailty is not restricted to follow a gamma distribution [33]. The proportional mean and rate model relaxed the non-homogeneous Poisson assumption for the counting process and directly models means and rates [17].

### Independent-increment model

The study question "Does the intervention decrease the event rate over the study period compared to the control?" is also addressed by Lin's independent-increment model for the rate of recurrent events [17]. Originally this model was developed by Andersen and Gill to specify the intensity of a counting process with a Cox-type link function [14]. Lin et al. provided a rigorous formalization of the marginal rate model, which relaxes the assumption that the event history, *F*_*i*_(*t*), can be completely described by time-dependent covariates, *x*_*i*_(*t*), that is, [17,30]

E[*dN*_*i*_(*t*)|*F*_*i*_(*t*)] = E[*dN*_*i*_(*t*)|*x*_*i*_(*t*)].

In contrast to Cox's model where subjects are at risk of an event until its occurrence or they are censored, in the independent-increment model subjects still remain at risk after an event occurs. Unlike the gamma-Poisson model, the independent-increment model does not assume the recurrent event rate is constant over time. This model assumes that the number of events in disjoint time intervals are independent [27].

Under the independent-increment model, the rate function, *λ*_*i*_(*t*), of any event for subject *i *is

(4) *λ*_*i*_(*t*) = Y_*i*_(*t*)*λ*_0_(*t*) exp{*βx*_*i*_(*t*)},

where

$${\text{Y}}_{i}(t)=\{\begin{array}{ll} 1, & \text{if subject }i\text{ is under observation at time }t\\ 0, & \text{if subject }i\text{ is censored by time }t. \end{array}$$

In model 4, Y_*i *_is the at risk indicator of event for subject *i*, *λ*_0_(*t*) is the baseline rate function for the event, *x*_*i *_is a covariate value, which may be time-dependent but may not contain elements of the event history, for subject *i*, *β *is the regression coefficient, and *t *represents the time from start of observation.

From model 4 we observe that both the baseline rate functions, *λ*_0_, and regression parameters, *β*, are assumed to be common across events.

Subjects are at risk of the an event until they are censored. Suppose *x*_*i *_is a binary indicator of group membership, with value 0 if subject *i *belongs to a control group and 1 if an intervention group. Then exp$\left(\hat{\beta }\right)$ estimates the common rate ratio of event for the intervention group relative to the control. The rate ratio is assumed to be constant over time and common across recurrent events. We interpret rate ratios less than 1 as indicating the overall rate of event in the intervention group is 100 [1 - exp$\left(\hat{\beta }\right)$]% lower than in the control. This model has a similar interpretation to the gamma-Poisson model except we no longer require the assumption of time-homogeneity or gamma distributed frailty.

Under the independent-increment model, the data for these subjects use the counting process format, where each subject is represented by a set of time intervals and event indicators. We illustrate these data in Table 2 using the example described in the Gamma-Poisson model subsection. In this data set, pid is the subject identifier, tstart is time of previous event or study start, tstop is time of event or censoring, status is an indicator of event, and grp is the covariate for group membership. Subject 1 experienced 3 events and then was censored at the end of follow-up, so there are 4 corresponding records for this subject. In contrast, subject 2 experienced 2 events before being censored, so there are only 3 records.

**Table 2:** Data structure for the independent-increment model

pid	tstart	tstop	status	grp
1	0	126	1	0
1	126	216	1	0
1	216	314	1	0
1	314	365	0	0
2	0	42	1	1
2	42	350	1	1
2	350	365	0	1

The corresponding SAS code to fit an independent-increment model is as follows:

PROC PHREG COVM COVS(AGGREGATE);

   WHERE (tstart < tstop);

   MODEL (tstart, tstop) * status(0) = grp/RISKLIMITS;

   ID pid;

RUN;

### Conditional models

RCTs on recurrent events provide insight into the study question "What is the effect of intervention on the rate of subsequent event among those who experienced the preceding event?", which a condtional model can address. Pepe and Cai proposed the conditional model for the rate of recurrent events, where subjects are not considered to be at risk for event until all previous events have occurred [18].

Under the total, follow-up time conditional model, the rate function, *λ*_*ij*_(*t*), of the *j*th event for subject *i *is

(5) *λ*_*ij*_(*t*) = Y_*ij*_(*t*) *λ*_0*j*_(*t*) exp{*β*_*j*_*x*_*i*_(*t*)},

where

$${\text{Y}}_{ij}(t)=\{\begin{array}{ll} 1, & \text{if }(j-1)\text{th event occured by time }t\text{ and }j\text{th event has not for subject }i\\ 0, & \text{if otherwise or censored at time }t\text{ for subject }i. \end{array}$$

From model 5 we observe that both the baseline rate functions, *λ*_0*j*_(*t*), and regression parameters, *β*_*j*_, can vary across events. The covariate *x*_*i *_may not contain elements of the event history.

In model 5, *t *represents the time from start of observation. The conditional model can also be formulated in terms of "gap time", the time from previous event:

(6) $$\lambda_{ij}(t-T_{N(t^{-})})={\text{Y}}_{ij}(t)\lambda_{0j}(t-T_{N(t^{-})})\exp\{\beta_{j}x_{i}(t)\},$$

where

$${\text{Y}}_{ij}(t)=\{\begin{array}{ll} 1, & \text{if }(j-1)\text{th event occured by time }t\text{ and }j\text{th event has not for subject }i\\ 0, & \text{if otherwise or censored at time }t \end{array}$$

and $T_{N(t^{-})}$ is the time of the event just prior to time *t*.

In contrast to the marginal model, subjects are considered at risk for an event at time *t *only if the previous event occurred before that time and they are still under observation. Suppose *x*_*i *_is a binary indicator of group membership, with value 0 if subject *i *belongs to a control group and 1 if an intervention group. Then, exp$\left({\hat{\beta }}_{j}\right)$ from model 5 estimates the event-specific rate ratio of the *j*th event from study start in the intervention group relative to the control, conditional on experiencing the previous events. The event-specific rate ratio for the *j*th event from model 6 represents the rate of the *j*th event from the time of the previous event in the intervention group relative to the control. We interpret rate ratios less than 1 as indicating that among those who experienced *j *- 1 events, the intervention reduces the rate of the *j*th event by 100[1 - exp$\left({\hat{\beta }}_{j}\right)$]% compared to the control. While the conditional model using total follow-up time compares subjects who experienced the same number of events and have the same follow-up from study start, the gap-time conditional model compares subjects who have experienced the same number of events and have the same duration since their previous event.

Fitting these conditional models relies on creating the appropriate data sets. These data sets are illustrated through the example presented in Gamma-Poisson model subsection. Under the conditional model for total follow-up, the data set for these subjects follows the counting process format as shown in Table 3. Similar to the independent-increment model (equation 4), the number of records representing each subject depends on the number of events experienced. The data structure differs from that of the independent-increment model since we have a variable for the event number.

**Table 3:** Data structure for the conditional model for total follow-up time

pid	tstart	tstop	event	status	grp
1	0	126	1	1	0
1	126	216	2	1	0
1	216	314	3	1	0
1	314	365	4	0	0
2	0	42	1	1	1
2	42	350	2	1	1
2	350	365	3	0	1

Assuming that the most number of events observed per subject was seven, the corresponding SAS code for fitting a conditional, total follow-up time model is as follows:

PROC PHREG;

   MODEL (tstart, tstop) * status(0) = group1-group7/RISKLIMITS;

      group1 = grp * (event = 1);

      group2 = grp * (event = 2);

      group3 = grp * (event = 3);

      group4 = grp * (event = 4);

      group5 = grp * (event = 5);

      group6 = grp * (event = 6);

      group7 = grp * (event = 7);

   STRATA event;

RUN;

Under the conditional, gap time model, the data set for these subjects requires times between adjacent events, as shown in Table 4. Again, the number of records per subject depends on the number of events experienced. As opposed to time intervals, times between subsequent events are required.

**Table 4:** Data structure for the conditional model for gap time

pid	gaptime	event	status	grp
1	126	1	1	0
1	90	2	1	0
1	98	3	1	0
1	51	4	0	0
2	42	1	1	1
2	308	2	1	1
2	15	3	0	1

Assuming that the most number of events observed per subject was seven, the corresponding SAS code for fitting a conditional, gap time model is as follows:

PROC PHREG;

   MODEL gaptime * status(0) = group1-group7/RISKLIMITS;

      group1 = grp * (event = 1);

      group2 = grp * (event = 2);

      group3 = grp * (event = 3);

      group4 = grp * (event = 4);

      group5 = grp * (event = 5);

      group6 = grp * (event = 6);

      group7 = grp * (event = 7);

   STRATA event;

RUN;

In these conditional model data sets, pid is the subject identifier, tstart is time of previous event or study start, tstop is time of event or censoring, gaptime is the time to event from previous event, event is the event number, status is an indicator of event, and grp is the covariate for group membership.

### Marginal model

"What is the protective effect of intervention on the rate of higher-order events compared to the control?" is an important study question to help decide whether to start treatment. This question is addressed by the marginal model, proposed by Wei, Lin and Weissfeld, which allows for different effects on each subsequent event [20]. This model treats the ordered event like an unordered competing risk problem [27]. Estimates from the marginal model have a practically useful interpretation which allows comparison between groups at treatment onset [34].

Under the marginal model, the rate function, *λ*_*ij*_(*t*), of the *j*th event for subject *i *is

(7) *λ*_*ij*_(*t*) = Y_*ij*_(*t*)*λ*_0*j*_(*t*) exp{*β*_*j*_*x*_*i*_(*t*)},

where

$${\text{Y}}_{ij}(t)=\{\begin{array}{ll} 1, & \text{if }j\text{th event has not occured by time }t\text{ for subject }i\\ 0, & \text{if otherwise or censored at time }t\text{ for subject }i. \end{array}$$

In model 7, Y_*ij*_, is the at risk indicator of the *j*th event for subject *i*, *λ*_0*j*_(*t*) is the baseline rate function for the *j*th event, *x*_*i *_is a covariate value, which may be time-dependent, for subject *i*, *β*_*j *_is the regression coefficient for event *j*, and *t *represents the time from start of observation. From model 7 we observe that both the baseline rate functions, *λ*_0*j*_, and regression parameters, *β*_*j*_, can vary across events.

Subjects are at risk of the *j*th event until it occurs or they are censored. Furthermore, subjects are considered to be at risk for the *j*th event even if they did not yet experience the (*j *- 1)th event. Suppose *x*_*i *_is a binary indicator of group membership, with value 0 if subject *i *belongs to a control group and 1 if an intervention group. Then, exp$\left({\hat{\beta }}_{j}\right)$ estimates the average event-number-specific rate ratio of the *j*th event in the intervention group relative to the control. We interpret rate ratios less than 1 as indicating the transition rate from 0 to *j *events in the intervention group is 100 [1 *- *exp$\left({\hat{\beta }}_{j}\right)$]% lower than in the control. The marginal event-number-specific rate ratios indicate whether subjects in the intervention group will have fewer higher-order events of a certain number from the time of treatment onset [34].

The data structure required for this model is illustrated through the example presented in the Gamma-Poisson model subsection. We would like to study the effect of intervention on the first four events. Under the marginal model, the data set for these subjects show times of event from study start for all events under study, as shown in Table 5. In this data set, pid is the subject identifier, tstart is time of study start, tstop is time of event or censoring, event is the event number, status is an indicator of event, and grp is the covariate for group membership. Both subjects are represented by the same number of records, namely four since we are interested in the first four events.

**Table 5:** Data structure for the marginal model

pid	tstart	tstop	event	status	grp
1	0	126	1	1	0
1	0	216	2	1	0
1	0	314	3	1	0
1	0	365	4	0	0
2	0	42	1	1	1
2	0	350	2	1	1
2	0	365	3	0	1
2	0	365	4	0	1

The corresponding SAS code to fit this marginal model is as follows:

PROC PHREG COVS(AGGREGATE);

   MODEL tstop*status(0)=group1-group4/RISKLIMITS;

      group1 = grp * (event = 1);

      group2 = grp * (event = 2);

      group3 = grp * (event = 3);

      group4 = grp * (event = 4);

   STRATA event;

   ID pid;

RUN;

## Results

Using available statistical instruments for recurrent events, we report results from a simple simulation study of falls prevention to illustrate the utility of the methods. Although each of the models being compared has already been studied via simulation, we contrast reporting results in the context of an RCT based on a single data set. The measures discussed are the rate ratios from the recurrent event models described in the Methods section. These include the common rate ratio, which compares the average rate of event in the intervention group to the control, the conditional event-specific rate ratios, which summarize the effect of intervention on a specific event conditional on experiencing previous events, and the marginal event-number-specific rate ratios, which summarize the intervention effect on the transition rate of experiencing a certain number of events from study start. In addition, we report the event rate, a measure of the average number of event accrued per person-time, and the mean cumulative function (MCF), a measure of the average number of events experienced per subject within a certain time.

We simulated recurrent falls in two groups, control and intervention, using Matlab Version 7 software (see Additional file 1). Each group had 250 subjects, and all subjects were followed for 365 days. Fall rates were based on those observed in an RCT [35]. Times between falls were assumed to follow an exponential distribution with falls rates specified for each fall. In the control group the fall rates for all falls were held constant at 7.7 falls per 1000 person-days. In the intervention group the fall rate was 5.3 falls per 1000 person-days for the first fall, and changed to 3.3 for all subsequent falls. Dependence within subjects was modelled using a gamma frailty distribution with density function given in equation 2 and variance *θ *= 0.10. We report the effect of the first 4 falls only since higher-order event-specific estimates are unreliable when there are only a few subjects with a large number of falls [25,27].

### Event rates

After 1 year, the control group had 675 falls, nearly double that of the intervention group with 373 falls. The total follow-up time in each group was 91,250 person-days. The average observed fall rates in the control and intervention groups were 7.4 (95%CI 6.8–8.0) and 4.1 (95%CI 3.7–4.5) falls per 1000 person-days, respectively. Compared to the control group, the rate of falls in the intervention was almost halved, a crude approximation of the anticipated effect size. This effect size can be used to design RCTs on recurrent events, specifically for determining the number of subjects.

### Mean cumulative function, MCF

**Figure 1:**
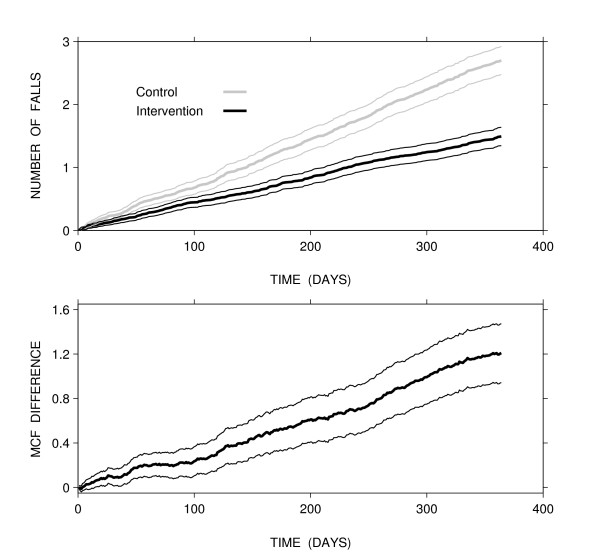
Estimated mean cumulative function (MCF) of falls by group (upper panel), their difference (lower panel), and 95% confidence intervals.

Figure 1 shows the MCF by group, estimated by a non-parametric estimator [36]:

$$\bar{\text{MCF}}(t)=\sum_{\left\{j|t_{j}\leq t\right\}}\frac{e_{j}}{n_{j-1}},$$

where *e*_*j *_is the number of events at time *t*_*j*_, *n*_*j*-1 _is the number of subjects at risk just beyond time *t*_*j*-1_, and *j *indexes the observed event times. A subject is at risk of event until the end of follow-up. At one year of follow-up, an average of 2.7 and 1.5 falls per subject were experienced in the control and intervention group, respectively. Both MCFs were approximately linear, which indicates that the rate of falls is relatively constant in each group [31,36]. The control group experienced more falls and had a higher fall rate than the intervention group. On average, the control group experienced 1 more additional fall by 301 days (Figure 1). From the MCF difference, we observed that 1.2 falls were prevented per year on average for each subject.

### Common rate ratios

The time-homogeneous gamma-Poisson and independent-increment gave similar common rate ratio estimates of 0.55 (95% CI 0.48–0.63) and 0.55 (95% CI 0.48–0.62), respectively (Table 6). The gamma-Poisson and independent-increment models both infer that the rate of any fall in the intervention group is 45% lower in the intervention group than control. In practice the assumption of a constant recurrent event rate over time may not hold, so the independent-increment model is preferred over the time-homogeneous gamma-Poisson model. These common rate ratios indicate that the intervention had an impact on the risk of falls; however, it does not inform whether the effect changes for subsequent events.

**Table 6:** Effect of intervention on recurrent falls, as measured by common rate ratios and 95% confidence intervals

Effect	Gamma-Poisson	Independent-increment
Control	1.00	1.00
Intervention	0.55 (0.48, 0.63)	0.55 (0.48, 0.62)

### Conditional event-specific rate ratios

The majority of the control group experienced two falls within 1 year of follow-up: 228, 180, 122, and 77 subjects had fall 1, 2, 3, and 4, respectively. The number of falls in the intervention group was lower: 202, 104, 45, and 18 subjects had fall 1 to 4, respectively (Table 7). Higher-order events, up to 7 falls, were experienced by 38 subjects in the control group; whereas, in the intervention group, only 4 subjects had the highest-order event of 5 falls. In the conditional model, the risk set for a subsequent fall consisted of only subjects who experienced the previous falls, and total follow-up time decreased for later events. The crude rate ratios indicate a similar intervention effect on falls 2 and 3.

**Table 7:** Fall-specific characteristics for total events, number of subjects at risk, total follow-up in days, and crude rate ratios, as indicated by the marginal and conditional total time models

Conditional model									
	Control				Intervention				
			
Event	# events	# at risk	follow-up	rate*	# events	# at risk	follow-up	rate*	crude RR^†^
fall 1	228	250	34,355	6.64	202	250	44,726	4.52	0.68
fall 2	180	228	24,361	7.39	104	202	30,264	3.44	0.47
fall 3	122	180	14,641	8.33	45	104	10,500	4.29	0.51
fall 4	77	122	9,673	7.96	18	55	4,301	4.19	0.53


Marginal model									
	Control				Intervention				
			
Event	# events	# at risk	follow-up	rate*	# events	# at risk	follow-up	rate*	crude RR^†^

fall 1	228	250	34,355	6.64	202	250	44,726	4.52	0.68
fall 2	180	250	58,716	3.07	104	250	74,990	1.39	0.45
fall 3	122	250	73,357	1.66	45	250	85,490	0.53	0.32
fall 4	77	250	83,030	0.93	18	250	89,791	0.20	0.22

* fall rate measured per 1000 person-days

^† ^RR = rate ratio

As expected, the rate ratios for the first fall from the conditional models give identical estimates, 0.68 (95% CI 0.57–0.83), since the total follow-up time and gap time to first falls refer to the same period (Table 8). For subsequent falls, the fall-specific rate ratios from the conditional models overlap and remain relatively constant ranging from 0.46 (95% CI 0.36–0.59) to 0.53 (95% CI 0.31–0.88). The rate ratio for fall 5, 0.38 (95% CI 0.13–1.07), may be unreliable due to the number at risk for this event, and effects could not be estimated for falls 6 or 7. Among subjects who experienced preceeding falls, the effect of intervention on the rate of the first four recurrent falls did not differ (Wald *χ*^2 ^test = 6.6, df = 3, *p *= 0.08 for total follow-up time model, and Wald *χ*^2 ^test = 6.7, df = 3, *p *= 0.08 for gap-time model).

**Table 8:** Effect of intervention on recurrent falls, as measured by fall-specific rate ratios and 95% confidence intervals

Effect	Conditional, total follow-up time*	Condtional, gap time^†^	Marginal^‡^
Control	1.00	1.00	1.00
Intervention			
fall 1	0.68 (0.57, 0.83)	0.68 (0.57, 0.83)	0.68 (0.57, 0.83)
fall 2	0.46 (0.36, 0.59)	0.46 (0.36, 0.59)	0.42 (0.33, 0.54)
fall 3	0.53 (0.38, 0.75)	0.53 (0.38, 0.75)	0.30 (0.21, 0.42)
fall 4	0.50 (0.30, 0.85)	0.53 (0.31, 0.88)	0.20 (0.12, 0.34)

*effects on recurrent falls were not different (*χ*^2 ^= 6.6, df = 3, *p *= 0.08)

^†^effects on recurrent falls were not different (*χ*^2 ^= 6.7, df = 3, *p *= 0.08)

^‡^effects on recurrent falls were different (*χ*^2 ^= 32.2, df = 3, *p *< 0.0001)

For recurrent falls, the rate ratios from the conditional, total follow-up time model indicate that conditional on experiencing the previous fall, the rate of second, third and fourth falls from study start are 54%, 47% and 50% lower in intervention than control. The rates of falls from the time of previous fall are 54%, 47%, and 47% lower in intervention than control, as estimated from the conditional, gap time model. The conditional models provide evidence of the constant difference in recurrent fall rates between the groups. The conditional fall-specific rate ratios evaluate how the intervention affected the rate of *k*th fall among those who experienced *k *- 1 falls.

For both the conditional total follow-up time model and conditional gap time model, subjects are considered to be at risk for an event only if the previous event occurred, so subjects at risk may not consist of all who were intially randomized. The number of subjects at risk for subsequent events should be reported to allow evaluation of how different the treatment groups are from the start of the study (Table 7).

### Marginal event-number-specific rate ratios

In the marginal model, all subjects were considered to be at risk for the 1st, 2nd, 3rd, 4th, and higher-order falls regardless of experiencing previous events (Table 7). Subjects are at risk for a specific fall until its occurrence or censoring, so the total follow-up time accumulates over subsequent falls. The crude rate ratios decrease with fall events.

The fall-number-specific rate ratios decrease from 0.68 (95% CI 0.57–0.83) for fall 1 to 0.20 (95% CI 0.12–0.34) for fall 4 (Table 8). For higher-order events, the rate ratio for fall 5 was 0.10 (95% CI 0.03–0.27) and could not be estimated for falls 6 or 7. The marginal model indicated that there was a difference in the average effect of intervention on the first four falls (Wald *χ*^2 ^test = 32.2, df = 3, *p *< 0.0001). Rate ratios based on the marginal model indicated that, on average, the transition rate from zero falls at the start of treatment to one, two, three and four falls were 32%, 58%, 70% and 80% lower, respectively, in the intervention group than the control. These rate ratios do not imply that the effect of intervention increased with recurrent falls. Rather, the marginal fall-number-specific rate ratios indicate that subjects in the intervention group will have fewer events overall.

Given an objective of an RCT is to compare groups which are similar in all aspects except for the treatment of interest, it is appropriate to use the marginal model since all subjects are considered to be at risk for each number-specific event from study start. In contrast, the groups being compared to evaluate the effect of subsequent events in the conditional models may not consist of all subjects initially randomized.

## Discussion

Recurrent events arise in many contexts, such as falls in seniors considered in this paper. In evidence-based medicine there is increasing need for guidelines on what to report in the analysis of recurrent events [8]. In the Results section we have outlined briefly statistical methods for evaluation of treatment effect from an RCT with a recurrent outcome. These should allow clinical researchers to report appropriate measures from an RCT for understanding the effect of intervention on the occurrence of a recurrent event.

We used a simulation study to relate an event process and results from analyses of the gamma-Poisson, independent-increment, conditional, and marginal Cox models [15-18,20]. We showed that each model has different study questions, assumptions, risk sets, and rate ratio interpretation, and so inferences should consider the appropriateness of the model for the RCT. The gamma-Poisson and independent-increment models compare the common event rates between groups, with the assumption of independence of the number of events across time intervals being required in the latter, but not the former. The conditional model distinguishes between first and recurrent events, and conditions on having had previous events. In contrast, the marginal model treats the events as unordered, and all subjects are at risk for any event. In different trials the outcomes of interest and validity of assumptions will differ. Our guidelines for reporting results from an RCT involving a recurrent event suggest statistical methods which correspond to the objectives of the trial, such as addressing the study question of interest, assessing comparable groups and estimating effect size. First, the average event rate by intervention group is a measure of the average number of events accrued per person-time. These event rates serve an important role in determining sample size and follow-up time for the design of future RCTs involving recurrent events [37]. Second, the MCF by intervention group provides a measure of the average number of events experienced per subject within a certain time. The MCF allows us to determine how many events per subject the intervention would prevent, on average, compared to the control group [31]. Third, the common rate ratio, as measured by the gamma-Poisson and independent-increment models, quantifies the average rate of event in the intervention group relative to the control group. This rate ratio provides an estimate of the common effect size, thereby indicating whether the intervention had an impact on the event occurrence. Fourth, conditional event-specific rate ratios, which quantify the rate of the *k*th event in the intervention relative to the control, conditional on experiencing preceding events, should be reported. These rate ratios allow us to evaluate how the effect of intervention changes, if at all, on subsequent events. Lastly, we suggest reporting the marginal event-number-specific rate ratios, which represent the rate of transitioning to higher-order events from the start of treatment in the intervention group relative to the control group. These rate ratios allow us to evaluate the overall protective effect of intervention. For methods used in the assessment of goodness of fit for each model we refer the reader to the corresponding papers [17,27].

It has been argued that the average event rate might have little relevance in the context of recurrent events because this measure does not acknowledge dependence between events experienced by a subject [38]. However, by applying appropriate statistical methods for recurrent events we can make valid inferences on rates. Extensive simulation studies based on varying event processes and case studies have compared recurrent event methods to determine their strengths and weaknesses [10-13].

Regression methods for the analysis of recurrent events is not limited to modelling the rate of event. The mean number of recurrences can be modelled using semi-parametric Cox models and parametric models [17,39]. Proportional rates and proportional means models are equivalent when the rate only depends on covariates that do not directly impact the occurrence of event, namely external covariates [17,40]. Regression models for the intensity function, which condition on event history, are also available [14,19]. However, in RCTs treatment may affect event history, so conditioning on the event history may underestimate the treatment effect [41].

## Conclusion

Our guidelines for reporting results from an RCT involving a recurrent event suggest that the study question and the objectives of the trial, such as assessing comparable groups and estimating effect size, should determine the statistical methods. Guidelines for reporting results from an RCT involving a recurrent event should allow clinical researchers to report appropriate measures for understanding the effect of intervention on the occurrence of a recurrent event.

## Competing interests

The authors declare that they have no competing interests.

## Authors' contributions

Study concept and design: BGS, LK. Analysis and interpretation: LK, BGS, MGD. Drafting of the manuscript: LK, BGS, MGD.

## Pre-publication history

The pre-publication history for this paper can be accessed here:



## Supplementary Material

Additional file 1 Matlab code to simulate recurrent falls data used in Results section Click here for file
